# MicroLet-7b Regulates Neutrophil Function and Dampens Neutrophilic Inflammation by Suppressing the Canonical TLR4/NF-κB Pathway

**DOI:** 10.3389/fimmu.2021.653344

**Published:** 2021-03-29

**Authors:** Binzhen Chen, Jia Han, Shaoheng Chen, Rufeng Xie, Jie Yang, Tongming Zhou, Qi Zhang, Rong Xia

**Affiliations:** ^1^ Department of Blood Transfusion, Huashan Hospital, Fudan University, Shanghai, China; ^2^ Blood Engineering Laboratory, Shanghai Blood Center, Shanghai, China; ^3^ Shanghai Key Laboratory of Data Science, School of Computer Science, Fudan University, Shanghai, China

**Keywords:** MicroLet-7b, neutrophil, inflammation, TLR4/NF-κB, sepsis, COVID-19

## Abstract

Sepsis is a heterogeneous syndrome caused by a dysregulated host response during the process of infection. Neutrophils are involved in the development of sepsis due to their essential role in host defense. COVID-19 is a viral sepsis. Disfunction of neutrophils in sepsis has been described in previous studies, however, little is known about the role of microRNA-let-7b (miR-let-7b), toll-like receptor 4 (TLR4), and nuclear factor kappa B (NF-κB) activity in neutrophils and how they participate in the development of sepsis. In this study, we investigated the regulatory pathway of miR-let-7b/TLR4/NF-κB in neutrophils. We also explored the downstream cytokines released by neutrophils following miR-let-7b treatment and its therapeutic effects in cecal ligation and puncture (CLP)-induced septic mice. Six-to-eight-week-old male C57BL/6 mice underwent CLP following treatment with miR-let-7b agomir. Survival (n=10), changes in liver and lungs histopathology (n=4), circulating neutrophil counts (n=4), the liver-body weight ratio (n=4–7), and the lung wet-to-dry ratio (n=5–6) were recorded. We found that overexpression of miR-let-7b could significantly down-regulate the expression of human-derived neutrophilic TLR4 at a post-transcriptional level, a decreased level of proinflammatory factors including interleukin-6 (IL-6), IL-8, tumor necrosis factor α (TNF-α), and an upregulation of anti-inflammatory factor IL-10 *in vitro*. After miR-let-7b agomir treatment *in vivo*, neutrophil recruitment was inhibited and thus the injuries of liver and lungs in CLP-induced septic mice were alleviated (p=0.01 and p=0.04, respectively), less weight loss was reduced, and survival in septic mice was also significantly improved (p=0.013). Our study suggested that miR-let-7b could be a potential target of sepsis.

## Introduction

In 2020, the world was challenged by the COVID-19 pandemic. Despite therapeutic innovations, the novel coronavirus has remained a crude contributor to the mortality rate and has severely endangered public health worldwide (1). Sepsis is defined as a heterogeneous syndrome associated with organ dysfunction (2). The pathogenesis and mechanisms involved in COVID-19 and sepsis both converge on a pivotal role played by the host inflammatory response. Thus, findings concerning sepsis may be useful for anti-inflammatory therapy for patients with COVID-19. Unfortunately, current therapeutic choices are limited and fail to reduce the mortality rate associated with sepsis (3); therefore, a better understanding of the mechanisms of sepsis and providing new targets are critical to improve public health.

Neutrophils are essential for innate immunity and inflammation (4–6). They are primary effectors in immune responses to resist pathogen invasion and are also central contributors of inflammatory reactions (7, 8). They have emerged as important early mediators of inflammatory injury in various tissues, including the skin, heart, joints, and liver (9–13). However, the mechanism by which neutrophils are involved in the regression of inflammation remains unclear and requires further exploration.

There are estimates that 30%–80% of human protein-coding genes are under the control of microRNAs (miRNAs) (14). miRNAs are non-coding small RNA molecules that typically suppress the translation of specific target mRNAs through partial complementarity (15, 16). miRNAs have been identified to be related to inflammation (17). They involve the fine-tuning and moderate suppression of target gene expression, yet master regulators that inhibit various genes in the same pathways (18, 19). MiR-let-7b is a cross-species miRNA, present in multiple genomic locations, including in 10 mature let-7 subtypes and 13 precursor sequences with the same seed sequence (20). MiR-let-7 levels have shown changes in some tumors (21). Gao et al. reported that miR-let-7b targeted TRAF6 in the inflammation response of kidney diseases (22). Reithmair et al. revealed that cellular miR-let-7b was down-regulated in septic shock patients (23). Moreover, restoring miR-let-7b levels reduced the production of IL-6 and TNF-α in neonatal monocytes on LPS stimulation (24). These features suggest that miR-let-7b may be an appropriate regulator for the duration and the magnitude of inflammation.

Toll-like receptors (TLRs) contribute to innate immune recognition of pathogens (25). TLR4 can be easily activated by lipopolysaccharide (LPS), and then triggers the production of proinflammatory mediators. TLRs are a typical innate immune receptor and neutrophils are typical innate immune cells, so the role of TLR4 in neutrophil function has been an area of growing interest (26, 27). Activation of TLR4 by neutrophils can cause the shedding of L-selectin, enhance phagocytosis, reduce chemotaxis, and initiate superoxide generation, and the production of many cytokines (28). Nuclear factor-kappa B (NF-κB) is also a transcriptional factor that regulates a series of genes essential for innate and adaptive immunity and inflammation (29, 30). P65 is a key activation subunit of NF-kB. The canonical NF-kB activation is controlled by its inhibitor, IkB, which blocks the translocation of NF-kB p65 subunit to the nucleus (31, 32). Previous studies have shown that the restoration of the miR-let-7 level can inhibit vascular inflammation mediators, including NF-κB (33). However, the regulatory mechanisms of miR-let-7b on TLR4/NF-κB signaling in neutrophils have not been clearly defined.

Our results suggest that miR-let-7b could regulate immunosuppression by targeting the neutrophilic TLR4/NF-κB signal during CLP-induced sepsis. These results reveal novel mechanisms of the involvement of miR-let-7b in neutrophilic inflammatory activity and provide valuable therapeutic targets for severe inflammation-driven diseases, including sepsis and the current COVID-19.

## Materials and Methods

### miRNA-Target Prediction and Pathway Analysis

TargetScan (http://www.targetscan.org/vert_71/) was used to predict interactions between miRNA and mRNA using bioinformatics. **Supplementary Table 1** outlines the direct targets of hsa-let-7b-5p miRNA. DIANA-mirPath v.3.5 (http://snf-515788.vm.okeanos.grnet.gr/) was used to analyze gene pathways (34) (**Supplementary Table 2**).

### Human Neutrophil Isolation and Coculture

Whole blood was collected from healthy donors from the Shanghai Blood Center, China. Informed consent was provided by donors according to the institutional ethical criteria. Neutrophils were purified from peripheral blood using the MACSxpress^®^ Neutrophil Isolation Kit (Miltenyi Biotec) according to the manufacturer’s guidelines. Red blood cells were lysed with water. Purity was more than 95% using anti-CD15(+) and anti-CD16(+) by flow cytometry carried out on a FACS Calibur cell analyzer (BD Biosciences). The recovery rate was 3 to 5 million neutrophils per milliliter of blood. The cell morphology was examined under a microscope and the cell preparations activated during the separation process were excluded. Cells were then cultured in RPMI 1640 supplemented with 10% fetal bovine serum (FBS, Sigma, USA) in an incubator with 5% CO2 at 37°C. MiR-let-7b mimics (100 nM), miR-let-7b inhibitor (100 nM), and miR-let-7b mimics negative control (NC, 100 nM) were introduced in cultures with human-derived neutrophils four hours after LPS (100 ng/mL) preconditioning, respectively. MiRNA inhibitor is a single-stranded RNA molecule that can specifically inhibit endogenous miRNA function after binding to mature miRNA. Cells were harvested for subsequent real-time quantitative polymerase chain reaction (RT-qPCR) assay and western blotting analysis after four hours of treatment, and cell culture supernatants were harvested at 10 hours for ELISA detection.

### Western Blotting

The lysates were obtained using RIPA buffer (Sigma, USA) premixed with a protease inhibitor cocktail kit (Thermo, USA). Nuclear proteins were extracted using a nuclear and cytoplasmic protein extraction kit (Sangon, China, #C510001). Cells were lysed for 30 minutes and then centrifuged for five minutes at 12,000 ×g at 4°C. Next, the supernatant containing protein was resolved on a 10% acrylamide‐bisacrylamide gel (EpiZyme, China, #PG112), and the proteins were transferred onto a 0.45‐μm PVDF membrane (Millipore, Germany, #IPVH00010). Membranes were blocked using protein-free rapid blocking buffer (EpiZyme, #PS108) and then were incubated with primary antibodies at 4°C overnight, followed by a 1-hour incubation with horseradish-peroxidase (HRP)-conjugated secondary antibodies at room temperature. The primary antibodies used were as follows: anti-TLR4 (1:2000, ABclonal, China, #A17436), anti-NF‐κB p65 (1:3000, Cell Signaling, USA, #8242S), anti-IkBα (1:2000, Proteintech, China, #10268-1-AP), anti-β-actin (1:1000, Beyotime, China, #AF0003), and anti-GAPDH (1:2000, Proteintech, #10494-1-AP). All experiments were performed in triplicate.

### Quantitative Real-Time Polymerase Chain Reaction

Total RNA was isolated from treated neutrophils using Trizol reagent (Invitrogen, USA) and miRNA was isolated with the mir-Vana™ miRNA Isolation Kit (Ambion). Total RNA was reverse transcribed into cDNA using a cDNA Synthesis Kit (Takara) according to the manufacturer’s instructions. The levels of miRNA were determined using Bulge-LoopTM miRNA RT-qPCR system (Ribobio, Guangzhou, China). Next, RT-qPCR was performed using targeted gene primers (BioTNT) following the manufacturer’s cycling parameters and run on an ABI Prism 7500 Sequence Detection System (Applied Biosystems) using TB Green MasterMix (Takara). The primer sequences used were GAPDH forward *5’- GGG AAG GTG AAG GTC GGA GT -3’*, GAPDH reverse *5’-GGG GTC ATT GAT GGC AAC A -3’;* TLR4 forward *5’-GCA CAT CTT CTG GAG ACG ACT -3’*, TLR4 reverse *5’-CAT CCT GTA CCC ACT GTT CCT -3’;* IL-6 forward *5’- CAC TGG TCT TTT GGA GTT TGA G -3’*, IL-6 reverse *5’- GGA CTT TTG TAC TCA TCT GCA C -3’;* IL-8 forward *5’- AAC TGA GAG TGA TTG AGA GTG G -3’*, IL-8 reverse *5’- ATG AAT TCT CAG CCC TCT TCA A -3’;* TNF-α forward *5’- TGG CGT GGA GCT GAG AGA TAA CC -3’*, TNF-α reverse *5’- CGA TGC GGC TGA TGG TGT GG -3’;*IL-10 forward *5’- GTT GTT AAA GGA GTC CTT GCT G -3’*, and IL-10 reverse *5’- TTC ACA GGG AAG AAA TCG ATG A -3’.* Relative fold changes in expression were calculated by normalizing to a housekeeping gene (GADPH) to adjust for loading variation. Primers of miRNAs (miR-let-7b-5p and U6) were designed by RIBOBIO Corporation (Guangzhou, China). U6 was used as the internal control. The miRNAs sequences are covered by a patent.

### Flow Cytometry

Human neutrophil purity, mouse neutrophil counts, and human TLR4 expression were both measured by flow cytometry. Cells were collected and centrifuged at 250 ×g for five minutes. After being resuspended in 100 μL binding buffer for cell purity and counts, human neutrophils were marked with 10 μL PE-conjugated CD15 (BD Biosciences, CA) and 10 μL APC-conjugated CD16 (BD Biosciences), and neutrophil counts were marked with FITC-conjugated CD11b, APC-conjugated Ly-6G and PE-conjugated Ly-6C (BioLegend, CA) for 20 minutes at room temperature. Then, 200 μL binding buffer was added and cells were evaluated by a fluorescence-activated cell sorting (FACS) Calibur device (BD Biosciences). Expression of TLR4 was evaluated by flow cytometry using Goat anti-TLR4 primary antibodies (R&D Systems, USA, #AF1478). Donkey anti-Goat IgG-AlexaFluor 647 (1:500, Absin, China, #abs20027) were used as secondary antibodies. Cells were measured by a FACS Calibur device (BD Biosciences). Cell populations were analyzed using the FlowJo software, v10.4.

### Enzyme-Linked Immunosorbent Assay

Human IL-6, IL-8, IL-10, and TNF-α levels in the culture supernatants were measured with enzyme-linked immunosorbent assay (ELISA) kits according to the manufacturer’s protocols (R&D Systems, USA). Mouse IL-6 and CXCL1 levels in the serum were measured with ELISA kits following the manufacturer’s instructions (Novus, USA).

### Immunofluorescence

Neutrophils were cocultured with FITC-conjugated miR-let-7b mimics in a six-well plate for 30 minutes and were then centrifuged at 250 ×g for five minutes. Cells were then fixed with 4% formaldehyde for 20 minutes and resuspended in 200 μL deionized H_2_O. A volume of five μL cell suspension was added to the gelatin-coated slide and smeared with a pipette tip. Samples were surrounded with a hydrophobic barrier using a Super Pap Pen (XLPCC, Japan, XL2001). Samples were blocked in a blocking buffer (Beyotime, China, #P0102) for 45 minutes. Slides were then incubated with 10 μg/mL of TLR4 primary antibodies (R&D Systems, USA, #AF1478) and normal goat IgG control (R&D systems, #AB-108-C) overnight at 2–8°C. Slides were washed two times using 1% PBS and then incubated with secondary antibodies (1:500, Absin, China, #abs20027) for one hour. Slides were then washed as described previously. DAPI counterstain was added, incubated two–five minutes at room temperature and coverslips were mounted. The TLR4-miR-let-7b localization was visualized using the inverted FV1000-IX81 microscope (Olympus, Japan). Images were captured at 100× and 180× objectives using the FV10-ASW software, v01.01.

### Cecal Ligation and Puncture (CLP) Mice Model

Six-to-eight-week-old male C57BL/6 mice were purchased from the Shanghai Jie Si Jie Laboratory Animal Ltd. Animal experiments were approved by the Institutional Animal Care and Use Committee of Huashan Hospital, Fudan University. Animal models of sepsis induced by cecal ligation and puncture (CLP) were performed on C57BL/6 mice according to Rittirsch et al. (35). Mice were randomly divided into four groups: (1) sham group, sham operation without treatment; (2) CLP group; (3) CLP + agomiR-Let-7b-5p group; (4) CLP + agomiR-NC group. Agomir NC and agomiR-Let-7b-5p (Ribobio, Guangzhou, China) were directly injected into the tail vein at the dose of 10 nmol per mouse suspended in 200 μL of saline, respectively. All mice in groups three and four were injected one hour before surgery, and all four groups of mice were sacrificed after 48 hours. Blood was collected 48 hours after surgery by retro-orbital bleeding. After euthanasia, the lower two-thirds of the right lung was ligated and the remaining parts were lavaged twice with one mL cold sterile PBS to harvest the bronchoalveolar lavage fluid (BALF).

The white blood cell (WBC) and polymorphonuclear (PMN) counts were performed using the auto hematology analyzer (Mindray, China). The pooled BALF was analyzed for pulmonary PMN counts by flow cytometry (C6 Accuri, BD Sciences). The ligated two-thirds of the right lobe of the lung were dissected, one-third was used for analyzing the lung wet-to-dry weight ratios (the wet weight of lung was measured using an electronic scale and was then dried in the oven at 70°C for 24 hours to determine the dry weight (36)) and for macroscopic observation, the other third was fixed in 4% paraformaldehyde for tissue histology. The liver was weighed to calculate the liver-to-body ratio (liver-to-body ratio=liver mass/body mass). To evaluate survival rates, mice were monitored every 12 hours. Survival was estimated from the time of CLP surgery.

### Histology and Immunohistochemistry

After harvesting of BALF, one-third of the right lobe of the lung and the whole liver of each mouse were both dissected and fixed in 4% paraformaldehyde for 24 h. Tissues were embedded in paraffin and 4-μm sections were cut. Hematoxylin and eosin (H&E) and immunohistochemistry (IHC)-stained with anti-CD11b rabbit polyclonal (1:500, Servicebio, China) sections were prepared using standard techniques. CD11b positive cells were quantified in five random fields (400× magnification) and imaged using a slide scanner microscope (Nikon 80i, Germany). The area occupied was analyzed by selecting brown areas using Image J software (NIH, USA).

### Statistical Analysis

Experiments were performed at least three times with consistent results. R (version 4.0.3) were applied to statistical analyses. The correlation analysis was performed using the “glm” function in R. The prognosis analyses were performed using the R package “survival” to generate the Kaplan–Meier (K-M) curves and to calculate the log-rank p-value. Error bars are represented as means ± standard deviations (SD). Significances of two-group comparisons were determined using a two-tailed Student’s t-test. Comparisons of significant differences of more than two groups were analyzed by one-way ANOVA. Results were considered statistically significant at p-value <0.05.

## Results

### TLR4 Was Identified as a Potential Target of MiR-let-7b in Neutrophils

**Figure 1:**
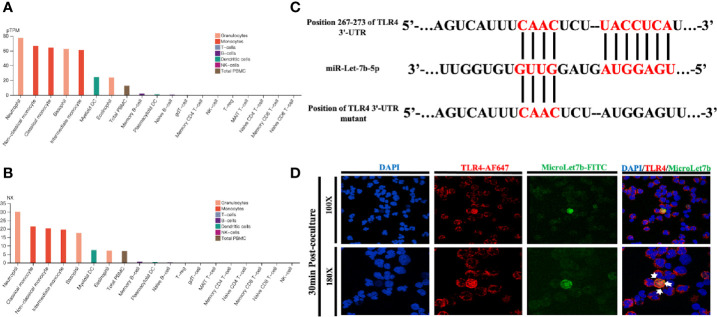
miR-let-7b directly targets TLR4 in neutrophils. **(A)** Expression profiles based on within-sample normalized pTPM for TLR4 gene enriched in 18 blood cell types and total PBMC ranked according to protein transcript expression values (see www.proteinatlas.org for details). **(B)** Same as **(A)**, but based on the between-sample NX values. **(C)** The sequence of human TLR4 3’-UTR (wild type and mutant) is predicted to be targeted by the mature sequence of miR-let-7b. Targeted nucleotides are labeled in red. **(D)** Confocal microscopy was performed on FITC-labeled miR-let-7b (green fluorescence) and cocultured for 30 minutes with AF647-labeled neutrophil TLR4 (red fluorescence) to demonstrate miR-let-7b internalization. Blue nuclear staining was performed with 4,6-diamidino-2-phenylindole (DAPI). One representative image of the immunostainings is shown. Scale bar denotes 100x and 180x objectives.

Using the data provided in the Human Protein Atlas (https://www.proteinatlas.org), a genome-wide transcriptomic depository of protein-coding genes in human blood cells (37), we first evaluated the presence of TLR4 expression (**Figure 1**) and the associated functional data of human neutrophils. Prior studies have shown that neutrophils could express TLRs 1, 2, 4, 5, 6, 7, 8, 9, and 10, which are essentially all the TLRs except for TLR3 (28). According to the analysis of the transcriptome, we determined the fraction of transcripts corresponding to different genes in each analyzed cell type and tissue. Thus, we reported the transcriptome usage for each representative blood cell types based on within-sample normalized pTPM values (**Figure 1A**) and between-sample normalized expression (NX) values (**Figure 1B**). The pTPM value was calculated by zooming in to a sum of 1 million TPMs of each sample to compensate for the previously deleted non-coding transcripts. The resulting transcriptional expression values, representing NX, were generated using 18 blood cell types and internal normalized channels of total PBMC. *In silico* analyses of the TLR and NF-κB signaling pathways indicated that miR-let-7b was among the top-ranked miRNA regulators of these two key inflammatory pathways (**Supplementary Figure 1** and **Supplementary Table 1**). The analysis showed that human miR-let-7b targets 15 and 14 genes within the TLR and NF-κB pathways, respectively. We found that TLR4 ranked highly in both pathways. Bioinformatics analysis identified that miR-let-7b could target TLR4 transcripts among the most significant regulators of the TLR signaling pathway (**Supplementary Figure 1** and **Supplementary Table 2**). The potential binding sites are shown in **Figure 1C**. Neutrophil extracellular traps (NETs) could be easily activated in sepsis or ALI (38, 39), thus we hypothesized that miRNA mimics, as is the case of bacteria, could target neutrophils in NETs-mediated sterile inflammation. After stimulation, resting neutrophils changed from sphere to oblate. Immunofluorescence colocalization analysis in neutrophils was used to examine the location and binding of TLR4 protein and FITC-conjugated miR-let-7b mimics. We found that miR-let-7b-FITC mimics were detectable within the neutrophil cytoplasm (nuclei stained blue DAPI) after 30 minutes of treatment (data showed in **Figure 1D**) without transfection. Consistent with the data analysis in **Figures 1A, B**, specific binding of AF647-conjugated TLR4 confirmed its high expression on the neutrophil cell membrane. Taken together, these data identified TLR4 as a direct target of miR-let-7b and miR-let-7b as an important regulator of inflammation-related activity.

### MiR-let-7b Activated the TLR4/NF-κB Pathway and Inhibited TLR4 Expression at the Post-transcriptional Level in Neutrophils

**Figure 2:**
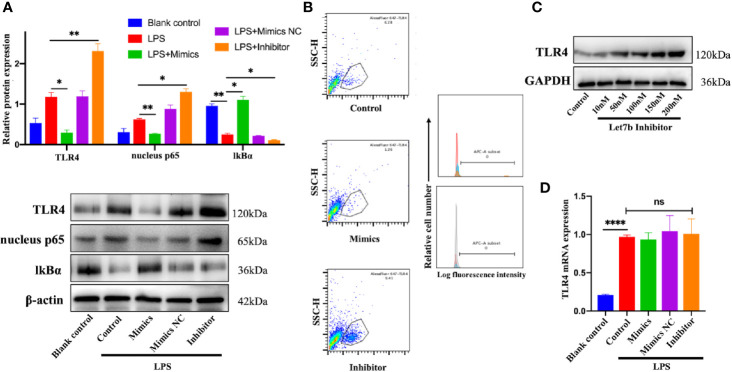
miR-let-7b activates the TLR4/NF-κB signaling and inhibits TLR4 expression *via* post-transcriptional regulation. **(A)** Neutrophils treated with 100 ng/mL LPS were then co-incubated with miR-let-7b mimics, miR-let-7b inhibitor, and miR-let-7b mimics NC (100 nM, respectively) for four hours followed by Western blotting to detect TLR4, nucleus p65, and cytosolic IkBα. *P < 0.05, **P < 0.01. **(B)** Isolated neutrophils were incubated with LPS alone or with indicated concentrations of miR-let-7b mimics and inhibitor (100 nM, each) for four hours followed by measurement of TLR4 binding (staining with AF647) by flow cytometry. The blue line is AF647-stained neutrophils cultured for four hours only (Blank), the red line is AF647-stained neutrophils pretreated with LPS and then cultured for four hours (Control), the brown line is AF647-stained neutrophils pretreated with LPS and then cocultured with miR-let-7b mimics for four hours (Let-7b mimics), and the gray line is AF647-stained neutrophils pretreated with LPS and then cocultured with miR-let-7b inhibitor for four hours (let-7b inhibitor). **(C)** A dose-dependent increase of TLR4 protein was determined with miR-let-7b inhibitor treatment. **(D)** RT-qPCR of miR-let-7b expression in human neutrophils. ****P < 0.0001, not significant (ns). All traces are representative of ≥3 independent experiments. Data represent mean ± SD (n=3).

To understand the mechanism responsible for miR-let-7b and TLR4-related inflammatory activity, we investigated the influence of miR-let-7b mimics (100 nM), miR-let-7b inhibitor (100 nM), and its negative control (NC, 100 nM), on TLR4 expression in neutrophils. IkB is a regulator of NF-kB upstream. It can bind to NF-kB and be degraded when the NF-kB signaling is activated. The expression level of IkB and nuclear NF-κB p65 can indirectly reflect NF-κB activation. As shown in **Figure 2A**, culture with miR-let-7b mimics resulted in a reduction in TLR4 and nuclear NF-κB p65 but increasing cytosolic IkBα protein expression (p<0.05, p<0.01, p<0.05, respectively), while treatment with the miR-let-7b inhibitor showed significant upregulation in TLR4 and nuclear NF-κB p65 but reduction in IkBα protein level (p<0.01, p<0.05, p<0.05, respectively). Similar results were also obtained using flow cytometry to detect cell surface expression of TLR4+ (**Figure 2B**). A lower percentage of TLR4+ (1.26%) was detected on the membrane of human neutrophils cultured with miR-let-7b mimics, whereas a higher percentage (9.41%) of TLR4+ cells on the membrane of human neutrophils cultured with miR-let-7b inhibitor were observed compared with control neutrophils (6.28%). In contrast, the miR-let-7b inhibitor increased TLR4 protein expression in a dose-dependent manner (**Figure 2C**). These results led us to ask whether miRNAs, which would influence both the translation and stability of mRNA, were involved in this process. But RT-qPCR showed that exposure to miR-let-7b exerted no significant impact on TLR4 mRNA levels (**Figure 2D**). These findings indicated that miR-let-7b could activate the TLR4/NF-κB pathway and inhibited TLR4 expression *via* post-transcriptional regulation in neutrophils.

### MiR-let-7b Mediated the Differential Effects on Secretion of IL-6, IL-8, TNF-α and IL-10 in Neutrophils

**Figure 3:**
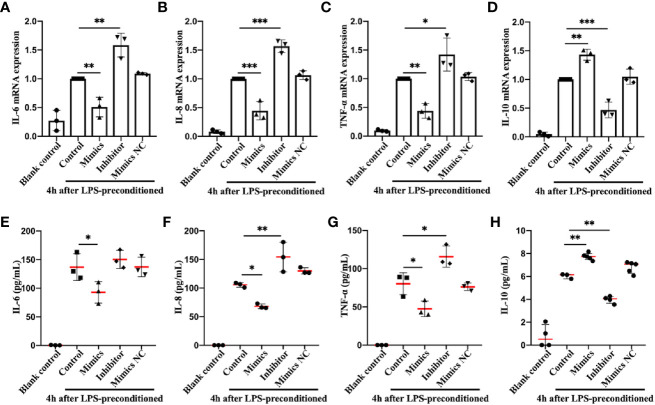
Identification and expression of inflammation-related cytokines in neutrophils *in vitro*. RT-qPCR **(A–D)** and ELISA **(E–H)** detection in the supernatant of IL-6, IL-8, TNF-α, and IL-10 in human freshly isolated neutrophils. Cells were incubated with miR-let-7b mimics, miR-let-7b inhibitor, and miR-let-7b mimics NC (100 nM, respectively), and the supernatant was collected at 10 hours after stimulation with 100 ng/mL LPS for 4 h. Data represent mean ± SD (n=3–5). Tukey’s multiple comparison tests were used to generate the P-values indicated in the figure. *P < 0.05, **P < 0.01, ***P < 0.001.

Infection can stimulate the expression of pro-inflammatory genes, such as IL-6, TNF-α, etc., which can effectively eliminate microorganisms, accelerate tissue repair, and secret IL-10 and other anti-inflammatory cytokines to alleviate the inflammatory reaction (40). If the pro-inflammatory cytokine levels are balanced with those of anti-inflammatory cytokines, then the internal microenvironment and homeostasis are maintained; otherwise, a systemic inflammatory response or anti-inflammatory syndrome may result (41, 42), which from a physiological standpoint, would be caused by excessive activation of the body’s preventative mechanisms, rather than the outcome of viral or bacterial infection. To directly assess the effects of miR-let-7b on the secretion of inflammatory-related cytokines, miR-let-7b mimics, inhibitor, and mimics NC were cocultured with human-derived neutrophils four hours after LPS preconditioning, respectively (**Figures 3A–H**). The neutrophils could be activated by LPS into a pro-inflammatory status. The RT-qPCR and ELISA results showed that after pre-treatment with LPS and incubation with miR-let-7b mimics, neutrophils dramatically produced significantly fewer pro-inflammatory cytokines, including IL-6, IL-8 and TNF-α and more anti-inflammatory cytokine, IL-10 at 10 hours. Conversely, the miR-let-7b inhibitor promoted IL-8 and TNF-α production in neutrophils, but only decreased IL-10 secretion. Taken together, we made the preliminary conclusion that miR-let-7b is a homeostatic regulator of anti-inflammatory activity.

### MiR-let-7b Exerted Anti-Inflammatory Effects Through Activating TLR4/NF-κB Pathway in CLP-Induced Septic Mice

**Figure 4:**
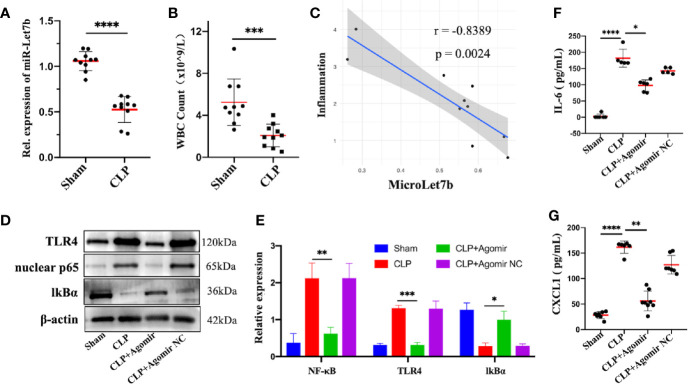
Overexpression of miR-let-7b suppresses neutrophilic inflammation *in vivo*. **(A)** Expression of miR-let-7b in sham and CLP-induced septic mice from the peripheral blood using RT-qPCR (n=10, ****P < 0.0001). **(B)** Total WBC count in sham and CLP-induced septic mice from the peripheral blood (***P < 0.001). **(C)** Correlation analysis between the level of miR-let-7b and the degree of septic inflammation (r=-0.8389, p=0.0024). **(D, E)** Effects of miR-let-7b agomir and its NC on neutrophilic TLR4, nuclear NF-κB p65 and IkB expression in CLP-induced septic mice were assessed by western blotting. *P < 0.05, **P < 0.01, ***P < 0.001. **(F, G)** Serum levels of IL-6 **(F)** and CXCL1 **(G)** were measured by ELISA in mice (n = 5-8 mice per group, *P < 0.05, **P < 0.01, ****P < 0.0001).

To investigate the role of miR-let-7b in inflammation *in vivo*, we performed the CLP surgery to establish a polymicrobial sepsis model. The expression of miR-let-7b was first detected in blood samples from sham and CLP-induced septic mice. We found that miR-let-7b expression was significantly decreased in septic mice compared with sham treated mice (p<0.0001, **Figure 4A**). Sepsis is a kind of severe inflammatory host response diagnosed with inflammatory variables like leukocytosis (WBC counts above 12,000/μL) or leukopenia (WBC counts below 4,000/μL) (43–45). The more severe the septic inflammation, the closer the disease to sepsis shock, the lower the WBC level. We, therefore, detected the circulating WBC and found that CLP-induced septic mice had leukopenia and WBC levels that were lower compared to the sham group (p<0.001, **Figure 4B**). Correlational analysis in **Figure 4C** showed that miR-let-7b negatively correlated with the degree of septic inflammation (r = -0.8389, p = 0.0024).

The critical role of miR-let-7b observed *in vitro* led us to speculate whether it is also involved in anti-inflammatory activity *in vivo*. As expected in western blotting analysis, TLR4 and nuclear NF-κB p65 expression increased while cytosolic IkBα decreased in CLP-induced septic mice, similar to that observed following exposure to miR-let-7b agomir NC, while overexpression of miR-let-7b by treatment with its agomir significantly decreased TLR4 and nuclear NF-κB p65 and increased IkBα protein levels in neutrophils (**Figures 4D, E**). Additionally, treatment with miR-let-7b agomir significantly decreased IL-6 and CXCL1 levels in mice serum relative to the CLP-treated group (**Figures 4F, G**). Thus, the inhibitory activity of miR-let-7b on CLP-induced pro-inflammatory cytokines secretion was attenuated with TLR4 and nuclear NF-κB p65 co-overexpression. Overall, these results demonstrated that miR-let-7b inhibited CLP-induced inflammation partly through the miR-let-7b/TLR4/NF-κB axis in neutrophils.

### Targeting MiR-let-7b Can Ameliorate Lungs and Liver Inflammation and Improve Survival in Sepsis Mice

**Figure 5:**
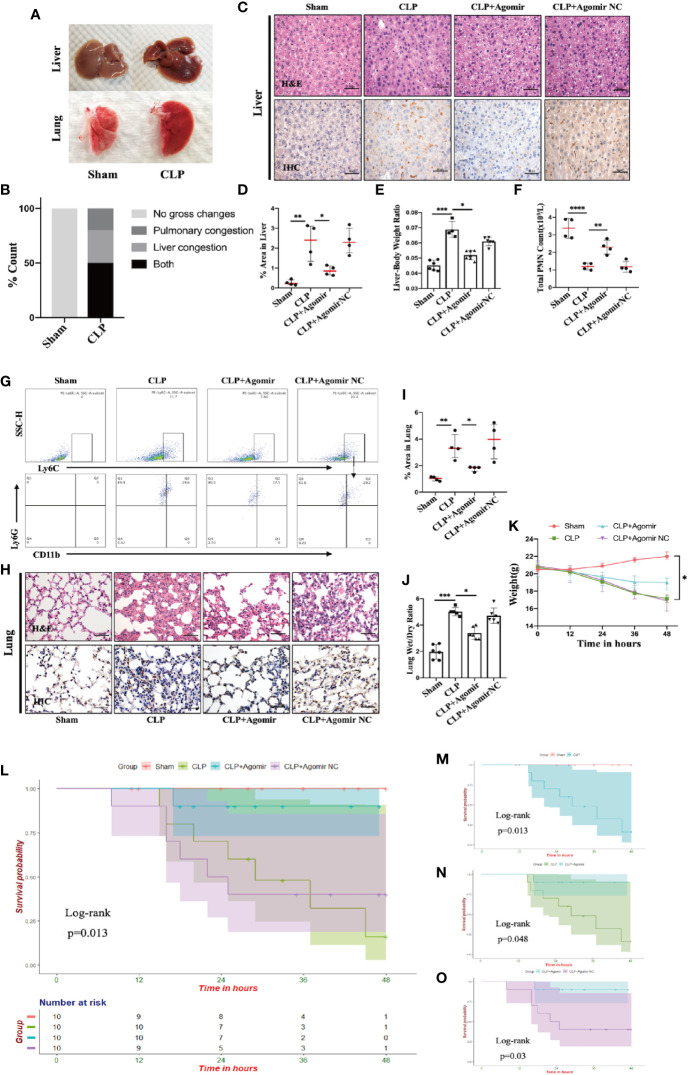
Targeting miR-let-7b alleviates tissue injury and improves overall survival in CLP murine models. **(A, B)** Gross pathologic changes in mouse livers and lungs showing pulmonary/liver congestion in sham and CLP-treated mice groups. **(C)** Histological assessment of livers harvested at 48 hours. Shown are representative H&E stain (upper panel) and IHC images (lower panel). Infiltrating neutrophils were identified by CD11b (brown), Scale bars, 50 μm. **(D)** Area of IHC positive for CD11b expressed as a percentage and assessed in five aleatory images. *P < 0.05; **P < 0.01, one-way ANOVA, n= 4/group. **(E)** The whole liver was dissected and weighed for determination of liver-body weight ratio (liver to body ratio=liver mass/body mass). n=4–7/group. **(F)** Total neutrophil counts in the blood. **P < 0.01, ****P < 0.0001, one-way ANOVA, n= 4/group. **(G)** Percentage of neutrophil infiltration in mice lung was detected in BALF. Representative flow cytometry plots of CD11b+ and Ly6G+ frequency (bottom row) among Ly6Cint cells (top rows). At the end of the experiment (48 h), the lower two-thirds of the right lung were dissected, of which one-third was for lung histology **(H, I**, same as liver**)** and the other one-third was weighed for the lung wet-to-dry weight ratio analyses **(J)** (n=5–6, one-way ANOVA, *P < 0.05, **P < 0.01). **(K)** The weight of C57BL/6 mice was monitored every 12 hours (*P < 0.05). **(L)** Survival rate was performed by Kaplan-Meier analysis among four groups. p = 0.013. Survival rates were also performed between sham and CLP group **(M)**, CLP and CLP + miR-let-7b agomir group **(N)**, and CLP + miR-let-7b agomir and CLP + miR-let-7b agomir NC group **(O)**. n=10 mice/group, Log-rank test, p = 0.013, p = 0.048 and p = 0.03, respectively.

Pulmonary swelling and liver hyperemia are well-established features of the CLP-induced inflammatory model (35). On the basis of this model, we found that more than 50% of CLP-treated mice had simultaneously exhibited visibly evident liver hyperemia and pulmonary edema compared to the sham group (**Figures 5A, B**). Our present data revealed miR-let-7b as a potential molecule involved in the inflammatory responses. To confirm that miR-let-7b exerts important anti-inflammatory activity, we observed both inflamed organs in the CLP-induced polymicrobial sepsis. Tissue damage and inflammation in the liver (**Figure 5C**) and lungs (**Figure 5H**) were assessed by H&E and IHC. IHC analysis was performed for CD11b, a neutrophil/macrophage marker, in representative cases on the same section exposed to H&E staining. As presented in **Figures 5C–E**, in both the CLP and agomir NC groups, prominent hepatic inflammation in the form of hepatic sinusoid adherence, shrinkage cracking, hepatic steatosis, vacuoles, and higher liver/body weight ratio (**Figure 5E**) was observed. A hallmark feature of inflammation is neutrophil recruitment from the blood to the inflammatory tissue (46, 47). The total neutrophil counts in **Figure 5F** showed that neutrophils in peripheral blood were significantly reduced upon CLP treatment. Results in **Figures 5D, F** thus mutually corroborated each other. MiR-let-7b agomir treatment produced a significant improvement in these liver injury parameters.

**Figure 6:**
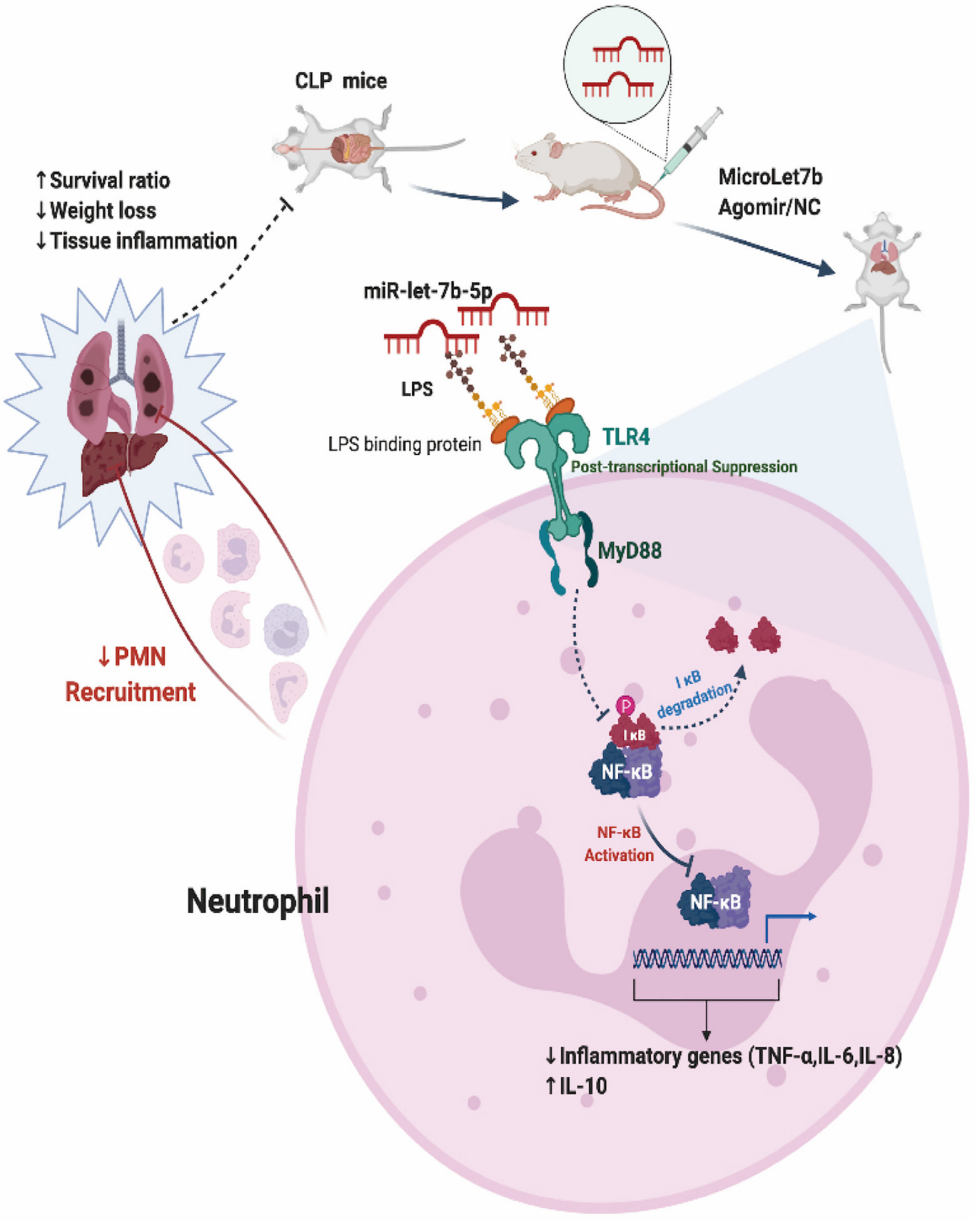
Proposed model for miR-let-7b-mediated regulation of neutrophilic functions that target TLR4/NF-κB axis *in vitro* and *in vivo*.

Sepsis is an infection-induced systemic inflammatory response syndrome (SIRS) with high morbidity and mortality (48). Sepsis patients are often characterized by lung function impairment (49). We next explored whether miR-let-7b could inhibit CLP-induced lung inflammation. In the BALF of CLP-induced septic mice, neutrophils were labeled with anti-Ly6G, anti-CD11b, and anti-Ly6C markers for flow cytometry analysis. As shown in **Figure 5G**, pre-treatment with miR-let-7b agomir attenuated lung neutrophilic inflammation, characterized by the reduced recruitment of neutrophils (stained with CD11b+, Ly6G+, and Ly6C int). An increased percentage (29.6%) of neutrophils (+) was observed in the BALF from lungs of CLP mice, whereas lower percentages (17.5%) of neutrophil (+) cells exposed to miR-let-7b agomir were observed compared with of baseline levels of miR-let-7b agomir-NC pre-injection (29.2%). Similarly, the CLP-induced mouse presented alveolar wall congestion, perivascular tissue edema, structural disorder, thickening of the alveolar septum, alveolar cavity narrowing, neutrophils infiltration, and higher lung wet/dry ratio (**Figures 5H, J**). In comparison, miR-let-7b agomir–treated mice significantly reduced these lung inflammatory responses, although it did not completely rectify them. In **Figures 5D, I**, CLP-induced septic mice exhibited an augmented positive signals indicative of CD11b expression (versus sham group), while miR-let-7b agomir treatment reduced this effect in both the liver and lungs (p=0.01 and p=0.04, respectively), further supporting the pivotal role of miR-let-7b during sepsis. It was worth mentioning that the total neutrophil counts in **Figure 5F** corroborated with these two results. During the modeling period, the body weights and survival status of all mice were measured every 12 hours. MiR-let-7b agomir treatment for 48 hours also resulted in reduced weight loss compared to both the CLP and CLP + agomir NC treatment (**Figure 5K**). Importantly, treatment with miR-let-7b agomir improved the survival rate (p=0.013) as evaluated using R software (survival package) (**Figure 5L**). CLP group mice showed worse survival compared to the sham group (**Figure 5M**). Significant improvements in overall survival were then observed in CLP and CLP + miR-let-7b agomir group (**Figure 5N**), and CLP + miR-let-7b agomir and CLP + miR-let-7b agomir NC group (**Figure 5O**). Altogether, these data pointed to a protective effect of miR-let-7b on CLP-mediated sepsis *in vivo*. A possible mechanism of miR-let-7b action *in vitro and in vivo* is indicated and outlined in **Figure 6**.

## Discussion

In the present study, we demonstrated that miR-let-7b could alter neutrophil function by suppressing the TLR4/NF-κB signaling pathway. Restoring miR-let-7b levels could effectively decrease levels of IL-6, IL-8, and TNF-α, while it increased IL-10 levels in freshly isolated human neutrophils. Treatment with miR-let-7b agomir could effectively protect mice from septic shock by reducing neutrophil recruitment into the liver and lungs. Here, we demonstrated that miR-let-7b expression was highly induced in an experimental murine model of sepsis. Given our findings, miR-let-7b is an attractive candidate for therapeutic strategies for severe inflammatory diseases.

Neutrophils are usually the first cells to reach the injured tissue, and thus may play an important role in the formation of the tissue inflammatory environment and exert profound effects on other somatic and immune cells. The migration of neutrophils into tissues is promoted by infection or sterile inflammation. After stimulation, circulating neutrophils leave the blood flow and accumulate at the site of infection or aseptic inflammation, where they release large numbers of highly toxic chemicals to engulf and kill pathogens. The classical recruitment of neutrophils from the blood into inflamed tissues is mediated by selectins and counter-receptors expressed on neutrophils (46). However, as Choudhury et al. reported in 2019 (50), neutrophil recruitment to the liver and lungs does not follow the classical cascade but require specific molecules.

Individuals with impaired neutrophils experience severe immunodeficiency both in quantity and effector functions (18). Immune cells can release a large number of proinflammatory cytokines, such as IL and TNF, to resist invasion or eliminate the pathogen. These cytokines will recruit additional immune cells in this battle until the immune system wins and achieves stability in the microenvironment. However, when the immune system is over-activated, the immune cells will overexpress cytokines, leading to the occurrence of a cytokine storm. Uncontrolled cytokine release will finally result in systemic inflammation, organ failure, and death. The cytokine storm may eventually lead to severe illness or death, in both sepsis and as observed in the current COVID-19 pandemic (51, 52). Furthermore, levels of IL-6 in severely ill patients were found to have increased significantly and were associated with a poor prognosis (53, 54). Corticosteroids and non-steroidal anti-inflammatory drugs are commonly used to treat inflammation. However, they do not alter the function of neutrophils or even enhance their destructive capacity (46). Excessive release of inflammatory cytokines leads to hyaline membrane formation, diffuse alveolar injury, and protein exudation to the lungs in COVID-19 patients, consistent with the pathological changes we observed in the lung of septic mice.

We have previously reported the therapeutic agents used for treatment of COVID-19 in China and found that the cytokine storm was not affected by neutralizing antibodies in convalescent plasma (55). Currently, different approaches to induce an anti-inflammatory response are being attempted to inhibit immune cell recruitment (allosteric antagonists to CXCR1/2 (56, 57)), release of pro-inflammatory factors (recombinant proteins or antibodies targeting IL-6, IL1-beta, IL-8, or IL-1 receptors), or inducing neutrophil degranulation (phosphodiesterase inhibitors) that have successfully reduced neutrophil recruitment in mice models. However, these findings relative to sepsis have not been translated into the clinic. No anti-inflammatory therapy has been effective in sepsis clinical trials and little is known about the role of miRNAs in regulating neutrophil function. An in-depth understanding of the miRNA function within neutrophils will help to identify potential clinical applications of miRNA as therapeutic agents. We thus developed various approaches including *in vitro* and *in vivo* experiments to determine whether let-7b levels could alter inflammation. Herein, we tested the therapeutic potential of restoring let-7b levels *in vitro* in human-derived neutrophils using let-7b mimics and *in vivo via* Let-7b agomir tail-vein injection in a CLP murine model.

Since primary neutrophils are granulocytes with a short lifespan, it is difficult to transfect them *in vitro*. Using a nucleofection plasmid transfection protocol, the transfection efficiency in neutrophils is low (~5%), when evaluated two hours post-transfection (58). Researchers have attempted to overcome this limitation by introducing granulocyte-macrophage colony-stimulating factor (GM-CSF) in the culture medium (59), while use of the leukemia cell line HL-60 as an alternative model (60) or direct electroporation of neutrophils (61) represent additional approaches to overcome this problem. The limitation for HL-60 cells is that they cannot mimic all aspects of neutrophil biology, and the conclusion from our *in vitro* experiment is that HL-60 cells cannot fully mimic the *in vivo* environments. As a result, we chose a mouse model to investigate the molecular mechanisms involved in regulating neutrophil function. Further, the extension of neutrophil lifespan by GM-CSF may alter its function and electroporation may also contribute to functional impairment. Since NETs may be easily induced when neutrophils are stimulated with LPS, likely through the activation of TLRs (62), and encouraged by experiments involving coculture of miRNA in activated platelet models (63, 64), we assumed that when neutrophils are cocultured with LPS and miRNA mimics, the former could induce NETs to capture the latter. We finally demonstrated that miR-let-7b mimics are introduced into the neutrophil cytoplasm after a 30-minute coculture following LPS stimulation (data shown in **Figure 1D**) without the need for transfection. To the best of our knowledge, this is an important technical achievement for introducing miRNA into neutrophils.

Previous studies have shown that a concerted action between different cells and the miR-let-7 family in the microenvironment is crucial for the outcome of the inflammatory response (20). For instance, exosome-derived let-7 can strongly suppress atherosclerotic inflammation (65). Let-7a can reduce the inflammatory response in microglia (66). Let-7g* can attenuate neuroinflammation by reducing microglia activation (67), and let-7d can suppress the atherosclerotic process in modulating PDGF and TNF-α signaling (33). However, not all miRNAs from the let-7 family benefit the vascular space. For instance, let-7a, b, e, and f aggravate neuronal damage following inflammation through TLR7 signaling (68). While, in neutrophils, the role of Let-7b is obscure. Thus, our research adds to the emerging body of evidence about the diverse impacts of inflammation-related members of the let-7 family.

Undeniably, our present study has several limitations to be considered that are mainly associated with its experimental design. Neutrophils are often described as “short-lived cells” with a life span of between 1.5 and 10 hours in mice and humans, which makes antimicrobial function studies and translation to clinical use a major challenge (46). Accordingly, we collected cell culture supernatants to be subjected to ELISA and cells for protein and mRNA detection within 10 hours of treatment. Further, our experiments could be performed under optimal conditions for neutrophil viability for up to 20 hours at 37°C in an anoxic culture medium supplemented with glucose and dimethyloxalylglycine (DMOG) (69) to validate the models *in vitro*. Moreover, our research lacks the recruitment of sepsis patients from clinical practice. Septic patients are usually treated with a combination of therapies at the time their samples and clinical details are collected. Thus, the effects of specific treatments concerning the expression levels of let-7b in sepsis patients could not be analyzed. Next, we only tested cytokines expression of IL-6 and CXCL1 in mouse serum samples as levels of TNF-α and IL-10 would be too low to be detectable by commercially available ELISA kits (data not shown). Another related problem is represented by our inability to present data relative to intravital imaging of neutrophils, which would provide more detailed behavior tracking of neutrophils under inflammatory condition, as this method is technically challenging. Future studies should be well designed and take these limitations into consideration.

Among miRNAs with altered expression in septic neutrophils, we demonstrate that miR-let-7b is involved in the regulation of genes related to inflammatory processes. Finally, biological therapies that improve miRNA processing should involve those that upregulate the levels of targeted miRNAs, which might contribute to suppress the activation of neutrophils. Thus, we strongly believe that in combination with the advances in miRNA delivery techniques, miR-let-7b will very soon be used to treat perilous human sepsis as well as the complications presented by the COVID-19 pandemic by regulating innate neutrophil function through modulation of the TLR4/NF-κB axis identified in this study.

## Data Availability Statement

The original contributions presented in the study are included in the article/**Supplementary Material**. Further inquiries can be directed to the corresponding author.

## Ethics Statement

This animal study was reviewed and approved by the Institutional Animal Care and Use Committee of Huashan Hospital, Fudan University.

## Author Contributions

BC, JH, and QZ designed the study. BC performed the experiments and wrote the manuscript. SC, RFX, and JY contributed analytic tools. BC and TZ analyzed the results. RX revised the manuscript and acquired the research funding. All authors approved the manuscript. All authors contributed to the article and approved the submitted version.

## Funding

This work was supported by the Chinese National Natural Science Foundation under Grant No. 82070197.

## Conflict of Interest

The authors declare that the research was conducted in the absence of any commercial or financial relationships that could be construed as a potential conflict of interest.

## Abbreviations

BALF, Bronchoalveolar Lavage Fluid; CLP, Cecal Ligation and Puncture; COVID-19, Corona Virus Disease 2019; LPS, Lipopolysaccharide; IL-6, Interleukin-6; IL-8, Interleukin-8; IL-10, Interleukin-10; miRNA, microRNA; NC, Negative Control; NETs, Neutrophil Extracellular Traps; NF-κB, Nuclear Factor Kappa-B; SIRS, Systemic Inflammatory Response Syndrome; TLR4, Toll-Like Receptor 4; TNF-α, Tumor Necrosis Factor-α.
