# Supplementary material for: MicroLet-7b Regulates Neutrophil Function and Dampens Neutrophilic Inflammation by Suppressing the Canonical TLR4/NF-κB Pathway
Source: Front Immunol. 2021 Mar 29;12:653344. doi: 10.3389/fimmu.2021.653344 (PMC8044834; doi:10.3389/fimmu.2021.653344)
Supplement: Supplementary file 1 [file DataSheet_1.pdf]

Supplementary Material

Supplementary Figure 1. Toll-like receptor signaling pathway components predicted to be regulated by hsa-let-7b-5p miRNA. Pathway analysis performed using DIANA LAB mirPATH v.3.(1) Predicted hsa-let-7b-5p targets are indicated by red star symbol.

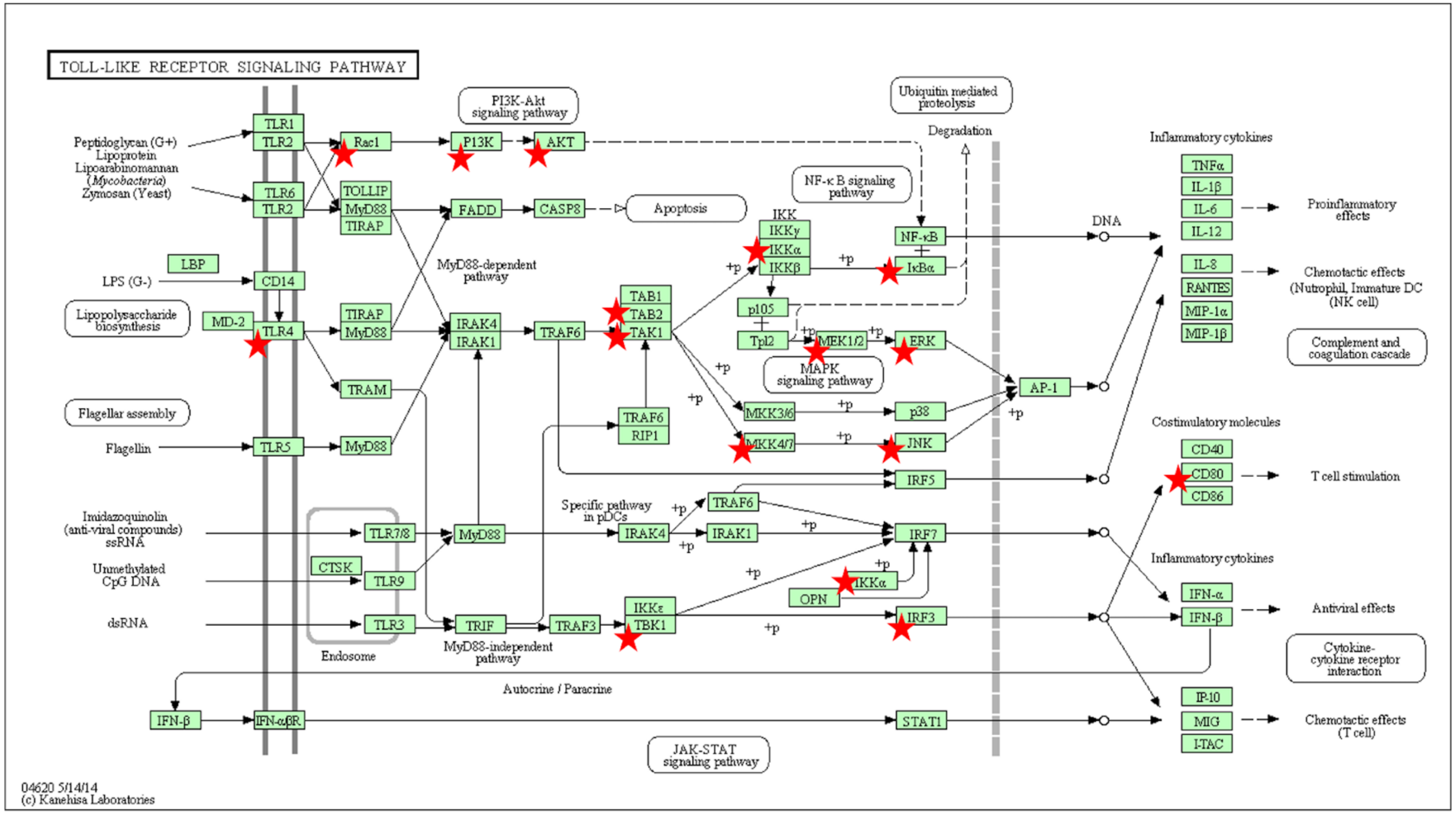

Supplementary Table 1. In silico prediction of top ranked miRNA-pathway interactions using Tarbase v7.0

		HUMAN				MOUSE		
Pathway	#Rank	miRNA	p-value	#genes targeted	#Rank	miRNA	p-value	#genes targeted
Toll-like receptor signaling pathway	1	hsa-miR-34a-5p	2.99965E-86	30	1	mmu-miR-26a-5p	2.777324e-44	16
	2	hsa-miR-522-5p	1.09899E-56	21	2	mmu-miR-128-3p	2.777324e-44	16
	3	hsa-miR-16-5p	1.9652E-50	19	3	mmu-miR-17-5p	2.777324e-44	14
	4	hsa-miR-17-5p	1.9652E-50	19	4	mmu-miR-27b-3p	5.751642e-38	14
	5	hsa-miR-107	2.35394E-47	18	5	mmu-miR-27a-3p	5.751642e-38	14
	6	hsa-miR-103a-3p	2.61151E-44	17	6	mmu-miR-26b-5p	5.751642e-38	13
	7	hsa-let-7a-5p	2.61151E-44	17	7	mmu-miR-22-3p	8.101307e-32	12
	8	hsa-miR-93-5p	2.67585E-41	16	…	…	…	…
	9	hsa-let-7b-5p	2.52411E-38	15	25	mmu-miR-31-5p	5.974400e-23	9
	10	hsa-miR-15b-5p	2.52411E-38	15	26	mmu-let-7b-5p	5.974400e-23	9
NF-kappa B signaling pathway	1	hsa-miR-34a-5p	7.858612e-84	28	1	mmu-let-7g-5p	3.846683e-55	19
	2	hsa-miR-522-5p	5.664637e-50	18	2	mmu-let-7i-5p	3.452951e-45	16
	3	hsa-miR-23b-3p	5.664637e-50	18	3	mmu-miR-27b-3p	6.008942e-42	15
	4	hsa-let-7a-5p	5.664637e-50	18	4	mmu-let-7c-5p	6.008942e-42	15
	5	hsa-let-7g-5p	9.090831e-47	17	5	mmu-let-7b-5p	6.008942e-42	15
	6	hsa-miR-16-5p	9.090831e-47	17	6	mmu-miR-27a-3p	6.008942e-42	15
	7	hsa-let-7f-5p	1.341387e-43	16	7	mmu-let-7f-5p	9.517234e-39	14
	…	…	…	…	8	mmu-let-7a-5p	9.517234e-39	14
	11	hsa-miR-15a-5p	2.240848e-37	14	9	mmu-miR-322-5p	9.517234e-39	14
	12	hsa-let-7b-5p	2.240848e-37	14	10	mmu-let-7e-5p	9.517234e-39	14

Supplementary Table 2. Tarbase Experimentally Supported Interactions for hsa-let-7b-5p in Toll-like receptor and NF-kappa B signaling pathway

	Toll-like receptor signaling pathway		NF-kappa B signaling pathway	
#	Gene Name	Gene Ensembl id	Gene Name	Gene Ensembl id
1.	MAP2K2	ENSG00000126934	CHUK	ENSG00000213341
2.	CD80	ENSG00000121594	TNFRSF11A	ENSG00000141655
3.	CHUK	ENSG00000213341	TLR4	ENSG00000136869
4.	TLR4	ENSG00000136869	TAB2	ENSG00000055208
5.	TAB2	ENSG00000055208	PLCG1	ENSG00000124181
6.	MAPK8	ENSG00000107643	NFKBIA	ENSG00000100906
7.	AKT1	ENSG00000142208	PTGS2	ENSG00000073756
8.	IRF3	ENSG00000126456	CSNK2A1	ENSG00000101266
9.	NFKBIA	ENSG00000100906	ERC1	ENSG00000082805
10.	TBK1	ENSG00000183735	PARP1	ENSG00000143799
11.	RAC1	ENSG00000136238	BIRC3	ENSG00000023445
12.	PIK3CA	ENSG00000121879	TRIM25	ENSG00000121060
13.	MAP2K4	ENSG00000065559	MAP3K7	ENSG00000135341
14.	MAPK1	ENSG00000100030	BIRC2	ENSG00000110330
15.	MAP3K7	ENSG00000135341		
